# Dominance of Cotton leaf curl Multan virus-Rajasthan strain associated with third epidemic of cotton leaf curl disease in Pakistan

**DOI:** 10.1038/s41598-024-63211-8

**Published:** 2024-06-12

**Authors:** Muhammad Arslan Mahmood, Nasim Ahmed, Athar Hussain, Rubab Zahra Naqvi, Imran Amin, Shahid Mansoor

**Affiliations:** 1 Agricultural Biotechnology Division, National Institute for Biotechnology and Genetic Engineering (NIBGE) College of Pakistan Institute of Engineering and Applied Sciences (PIEAS), Faisalabad, 38000 Pakistan; 2 Present Address: Plant Sciences Division, Research School of Biology, The Australian National University, Canberra, ACT 2601 Australia; 3 Department of Biological Sciences, University of Sialkot, Sialkot, 51310 Pakistan; 4 Biotechnology and Microbiology Group, Department of Zoology, University of Poonch Rawalakot, Rawalakot, Azad Jammu and Kashmir Pakistan; 5 Department of Biotechnology, Mohi-ud-Din Islamic University, Nerian Sharif, Azad Jammu and Kashmir Pakistan; 6 School of Food and Agricultural Sciences (SFAS), University of Management and Technology (UMT), Lahore, 54000 Pakistan; 7 International Center for Chemical and Biological Sciences, University of Karachi, Karachi, Pakistan

**Keywords:** Geminivirus, Cotton leaf curl Multan virus-Rajasthan strain, Southern Punjab and Sindh, Genetic diversity, Biotechnology, Evolution, Genetics, Microbiology, Plant sciences

## Abstract

Cotton (*Gossypium hirsutum*) is an economically potent crop in many countries including Pakistan, India, and China. For the last three decades, cotton production is under the constant stress of cotton leaf curl disease (CLCuD) caused by begomoviruses/satellites complex that is transmitted through the insect pest, whitefly (*Bemisia tabaci*). In 2018, we identified a highly recombinant strain; Cotton leaf curl Multan virus-Rajasthan (CLCuMuV-Raj), associated with the Cotton leaf curl Multan betasatellite-Vehari (CLCuMuB^Veh^). This strain is dominant in cotton-growing hub areas of central Punjab, Pakistan, causing the third epidemic of CLCuD. In the present study, we have explored the CLCuD diversity from central to southern districts of Punjab (Faisalabad, Lodhran, Bahawalpur, Rahimyar Khan) and the major cotton-growing region of Sindh (Tandojam), Pakistan for 2 years (2020–2021). Interestingly, we found same virus (CLCuMuV-Raj) and associated betasatellite (CLCuMuB^Veh^) strain that was previously reported with the third epidemic in the central Punjab region. Furthermore, we found minor mutations in two genes of CLCuMuV-Raj *C4* and *C1* in 2020 and 2021 respectively as compared to its isolates in 2018, which exhibited virus evolution. Surprisingly, we did not find these mutations in CLCuMuV-Raj isolates identified from Sindh province. The findings of the current study represent the stability of CLCuMuV-Raj and its spread toward the Sindh province where previously Cotton leaf curl Kokhran virus (CLCuKoV) and Cotton leaf curl Shahdadpur virus (CLCuShV) have been reported. The findings of the current study demand future research on CLCuD complex to explore the possible reasons for prevalence in the field and how the virus-host-vector compatible interaction can be broken to develop resistant cultivars.

## Introduction

Cotton (*Gossypium* spp.) stands among the leading natural fiber crops that not only produce fiber for textiles but also contributes significantly to livestock feed and edible oil. Pakistan is the 5th largest cotton producer of the world after India, China, the USA, and Brazil^1^. In South Asian countries, the cotton crop is facing multiple abiotic and biotic stresses. Among the biotic factors, cotton leaf curl disease (CLCuD) is one of the most notorious diseases and a real threat to the sustainable production of cotton in India and Pakistan^2^. CLCuD is caused by whitefly (*Bemisia tabaci*) transmitted viruses that are circular single-stranded (ss) DNA viruses affecting a wide host range of plants^3^ and classified into the genus *Begomovirus* and family *Geminiviridae* ^4^.

Begomoviruses are subdivided into monopartite, and bipartite, based on their genomic organization. The monopartite genome and DNA-A genome of bipartite viruses can encode six different proteins of which four proteins are on the complementary strand while two proteins are on the virion strand. The proteins encoded on the virion strand are (A)V1 (coat protein) and (A)V2 (pre-coat protein) while the proteins encoded on the complementary strand are (A)C1 (replication-associated protein), (A)C2 (transcriptional activator protein), (A)C3 (replication enhancer protein), and the (A)C4 protein. Moreover, additional small proteins (size range 5–10 kDa) can also be found in the tomato yellow leaf curl virus (TYLCV), a widespread monopartite begomovirus. This identified new protein (V3) act as a suppressor of RNA silencing^5^. The C4 protein of monopartite viruses and its bipartite homolog AC4 plays a vital role in virus pathogenicity. Disruption of the C4 protein reduces the symptom development in the infection cycle of several begomoviruses^6^. The DNA-B genome of bipartite viruses encodes two distinct proteins i.e. the movement protein (MP) and nuclear shuttle protein (NSP) in the complementary and virion strand, respectively^7^. The Begomovirus intergenic region is composed of cis-regulatory elements for the expression of genes and iterons (small repeated sequences) that have sequence-specific binding sites for the C1 protein^8^. It also contains the predicted hairpin structure that consists of the nonanucleotide (TAATATTAC) which is conserved in the geminiviruses. Both hairpins and iterons can make the origin of replication (ori) for viral replication. Bipartite viruses also share a “common region” that plays a vital role in sustaining split genome integrity by ensuring that the Rep protein is encoded by DNA-A component which initiates the virion strand replication for both components^9^.

**Figure 1:**
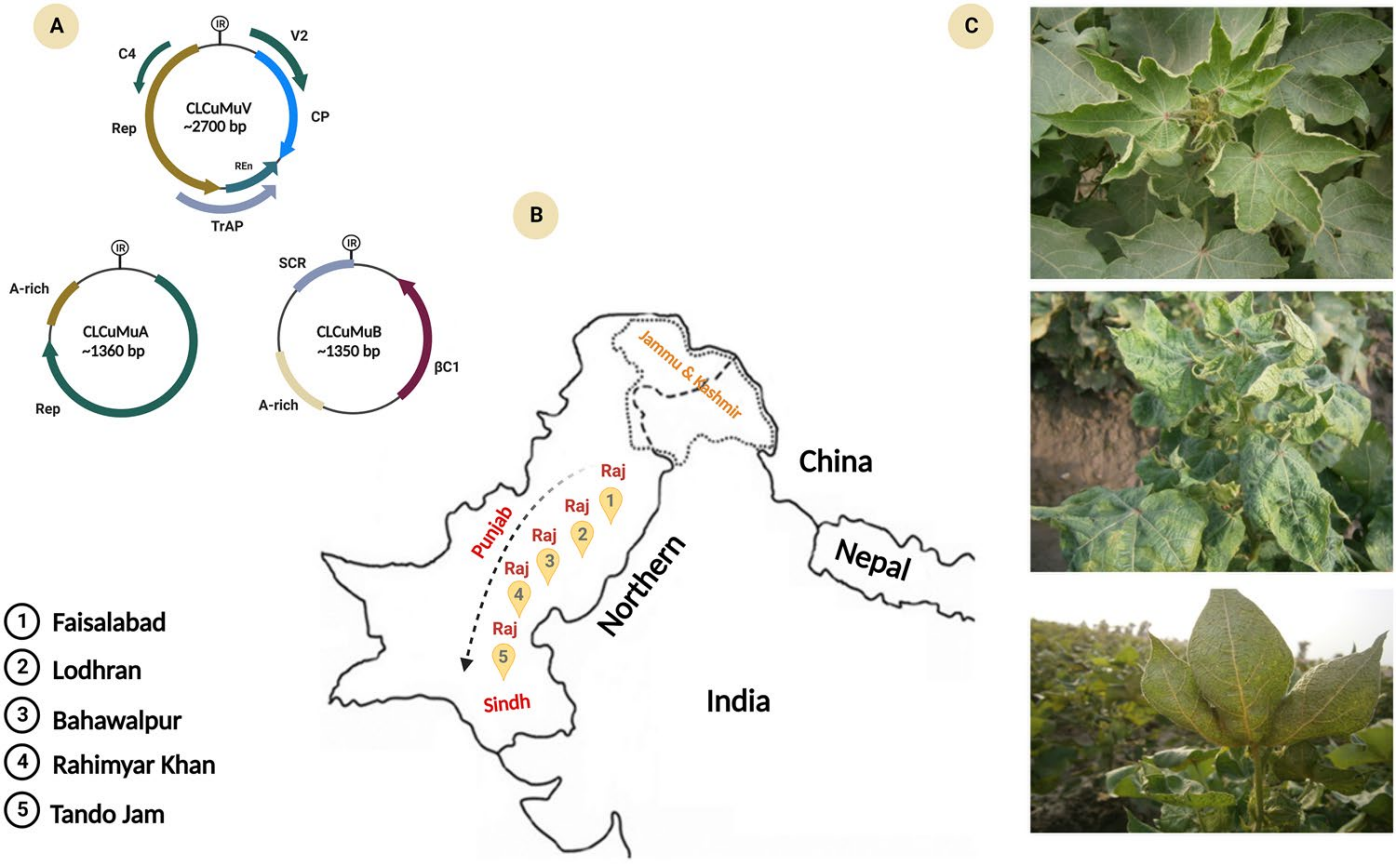
Genome organization and the geographical distribution of CLCuMuV-Raj and associated satellites. (**A**) CLCuMuV has a genome of ~ 2700 bp having two genes on virion strand encodes CP and MP protein while four ORFs on the complementary strand encodes Rep, TrAP, REn, and C4 proteins. Alphasatellites (CLCuMuA) and betasatellites (CLCuMuB) possesses genomes of ~ 1360 bp and ~ 1350 bp, respectively. (**B**) Distribution CLCuMuV-Raj strain in Punjab districts (1, 2, 3, and 4) and Sindh district (5) of Pakistan. (**C**) The typical symptoms of CLCuD, upward leaf curling, vein thickening, and leaf yellowing. The map was generated using CorelDRAW (version number 12.0) software (https://www.coreldraw.com/en/) and edited in Paint 3D and Microsoft PowerPoint 2019.

Small ssDNA helper molecules are associated with monopartite viruses referred to as alphasatellites, betasatellites, and deltasatellites^10^ (Fig. 1A). Betasatellite (previously known as DNA-β) is half of the size of begomovirus (~ 1350 base pairs) and encodes a single gene, βC1, of ~ 118 amino acids in the complementary sense. Betasatellites help in the development of disease symptoms and may also help in the accumulation of helper begomovirus in some host crops^11,12^. Alphasatellites (previously known as DNA-1) are not true satellites as they can independently replicate inside the host plants. They are dependent on the helper begomovirus for encapsidation, insect transmission and their movement within the plant^13,14^. Alphasatellites are also discovered in New World (NW) combined with bipartite begomoviruses, where betasatellites are not found^15,16^.

CLCuD was first reported from the southern Asia region in the 1960s and passed through four distinct phases i.e., pre-epidemic, epidemic, resistance breaking (also known as the second epidemic), and post-resistance breaking. From each of the phases, different reports were available describing the association of distinct viruses but the presence of single betasatellite species i.e., cotton leaf curl Multan betasatellite (CLCuMuB) was ubiquitous^2^. The first epidemic (Multan epidemic) was originated in 1990 from Multan, Khanewal, and Vehari and was associated with multiple begomoviruses such as Cotton leaf curl Multan virus (CLCuMuV), Cotton leaf curl Kokhran virus (CLCuKoV), Cotton leaf curl Alabad virus (CLCuAlV), and Papaya leaf curl virus (PaLCuV)^17^ along with a single betasatellite species, CLCuMuB^2^. Cotton breeders developed CLCuD-resistant varieties through conventional breeding and it led to the ending of Multan epidemic phase^18^. The second epidemic (Burewala epidemic) started at Burewala, Vehari district of Punjab, Pakistan in the early 2000s when the recombinant strain of CLCuKoV and CLCuMuV appeared i.e. CLCuKoV-Burewala (CLCuKoV-Bu). This recombinant virus was also associated with the recombinant version of CLCuMuB with most of its sequence from CLCuMuB but also comprising a small fragment of satellite conserved region (SCR) derived from tomato leaf curl betasatellite (ToLCB)^19^. This resistance-breaking recombinant virus (CLCuKoV-Bu) had a truncated C2 protein of only 35 amino acids (aa)^20,21^. But it was completed after some years and has also been identified showing severe symptoms of CLCuD^19^. The post-resistance breaking phase has much evidence that a slow shift from CLCuKoV-Bu to an epidemic phase with some earlier virus species/strains reappearing in cotton^22^. Other begomoviruses and mastreviruses (that are transmitted by leafhoppers) were also occasionally identified from cotton during all the phases from the past few years and CLCuMuB was also associated with the disease that is required for the development of the symptoms^23,24^. In 2015 and 2017, two studies from India and Pakistan were carried out independently on CLCuD in which Datta et al. detected the recombinant and phylogenetically distinct clade of CLCuMuV from infected cotton fields of Punjab, India. Moreover, they could not detect the CLCuKoV-Bu which was prevalent in the Indian subcontinent of almost a decade^25^. On the other hand, in Pakistan, Zubair et. al. showed the presence of multiple begomoviruses (CLCuMuV, CLCuKoV, and CLCuAlV) that were found in the first epidemic in the 1990s^26^. Later Ahmed et al. precisely identified the CLCuD complex by employing the new sequencing method CIDER-Seq (Circular DNA Enrichment Sequencing) and identified a single dominant strain of CLCuMuV i.e. a novel variant of the Rajasthan strain that developed the distinct clade with some other recent isolates reported after 2015 both from India and Pakistan, distinct CLCuMuB-Vehari strain and multiple species of alphasatellites^27^. Begomoviruses often undergo recombination, thus increasing the chances of diversity.

In this study, we explored the CLCuD complex from cotton-growing areas of central to southern Punjab and Sindh districts of Pakistan and found that CLCuMuV-Raj is the most prevalent strain of all over the Punjab and it has also moved toward the Sindh province of Pakistan where previously CLCuKoV and cotton leaf curl Shadadpur virus (CLCuShV) have been dominant. Furthermore, we also identified C4 and C1 mutation from CLCuMuV-Raj sequences found in Punjab province but have not found any mutation in CLCuMuV-Raj sequences from Sindh. Results revealed that CLCuMuV-Raj is the most dominant strain in Pakistan as well as in the northwestern zone of India, this strain is now recognized as a major strain associated with the third epidemic of CLCuD.

The purpose of the current study was to determine the genetic diversity of begomoviruses/satellites associated with CLCuD in the years 2020–21 with extended regions. To control the begomoviruses and to develop plant virus resistance strategies, knowledge about viral diversity and their classification is very important.

## Materials and methods

### Collection of CLCuD-infected cotton leaf samples

Field surveys were conducted in 2020 and 2021 to estimate the CLCuD incidence in southern Punjab and Sindh provinces of Pakistan (Fig. 1B). The southern Punjab districts including Faisalabad (FSD), Bahawalpur (BWP), Lodhran (LOD), Rahimyar Khan (RYK), and in Sindh province district Tandojam (TJ), were selected for the survey based on the major cotton growing areas. We conducted a survey of 15 cotton fields and collected 30 leave samples that were infected with severe symptoms of CLCuD such as leaf curling (upward and downward), leaf enation, yellowing, and vein thickening (Fig. 1C). We collected four symptomatic and one asymptomatic leaf from each field and were placed in labeled polythene bags followed by transport to the laboratory. Thirty leaf samples (2 taken from each of the 15 sampled field) were used for DNA extraction and further processing. The quality and quantity of the extracted DNA were checked on agarose gel electrophoresis and by spectrophotometer respectively.

### Extraction of genomic DNA and viral genome enrichment

The total genomic DNA of symptomatic and asymptomatic leaves was extracted^28^. The DNA quality was checked by Nanodrop Spectrophotometer. Then, circular viral/satellite DNA enrichment was performed by the phi29 DNA polymerase mediated rolling circle amplification (RCA) method using^29^ and following the manufacturer’s instructions (Thermo Fischer Scientific, Waltham, MA, USA).

### Cloning of begomovirus/satellites and their Sanger sequencing

The full-length virus and its satellites isolated from infected cotton samples were selected for cloning and sequencing. The RCA product was diluted (50–100 ng/µL) and used in a polymerase chain reaction (PCR) using begomovirus^30^, alphasatellites^31^, and betasatellites universal primers^32^. Then the product was cleaned and cloned in PTZ57R/T^33^. The positive confirmed clones obtained were sequenced by using M13 forward and reverse primers on ABI 3730XL DNA sequencer (USA). Another set of primers was designed using primer walking strategy for achieving sequence of complete genome of begomoviruses.

### Sanger sequence reads assembly, ORF and BLASTn analysis

DNASTAR Lasergene’s SeqMan Ultra (version number 5) software (Madison, Wisconsin, USA; https://www.dnastar.com/software/lasergene/) was used to assemble the sequenced reads. A single consensus contig was analyzed after the trimming of vector portion and the sequence was searched in the NCBI database by BLASTn (https://blast.ncbi.nlm.nih.gov). The coding regions of viruses and their associated satellites were identified using ORF finder (https://www.ncbi.nlm.nih.gov/orffinder/).

### Sequence demarcation tool analysis for species and strain analysis

Sequence demarcation tool (SDT) was used to perform analysis for species and strain demarcation by using the following taxonomic criteria of begomovirus i.e., 94% and 91% pairwise sequence identity (PSI) for strain and species, respectively^34,35^. For alphasatellites, 70% and 88% PSI for genus and species demarcation^36^ while for betasatellites, previously 78% PSI score was recommended^37,38^ but recently Bridden et al*.* proposed 91% PSI score for betasatellite demarcation.

After demarcation, alignment of these newly isolated begomovirus genomes and associated satellites was done through MUSCLE by using default settings of SDT with top similar sequences (retrieved by BLASTn) available on NCBI.

### Phylogenetic analysis of begomovirus and their associated satellites

Molecular Evolutionary Genetics Analysis (version number 7.0) (MEGA7) software (https://www.megasoftware.net/) was used for the phylogenetic analysis of the begomoviruses, and associated satellites identified in the current study. MUSCLE was utilized for multiple sequence alignment available in MEGA7^39,40^. To draw inferences about the relatedness among isolated begomoviruses in the present study with previously reported isolates, a phylogenetic tree was re-constructed by using the neighbor-joining method with bootstrapping test of 1000 replications^41,42^. The full genome sequences of the CLCuMuV-Raj strain were retrieved from NCBI. One sequence of papaya leaf curl virus (PaLCV: AJ436992) was taken as an outgroup. Similarly, alphasatellites (used the 55 reference geminialphasatellites species sequences given by Varsani et al. ^38^ and betasatellites sequences (sequences of different strains of CLCuMuB) were also phylogenetically analyzed.

### Mutation detection and protein modeling of C1 and C4 protein of CLCuMuV-Raj strain

The adaption and evolvability of DNA viruses majorly depend on recombination events and minor on the assimilation of genome-associated changes. The mutation in C1 and C4 protein was determined through multiple sequence alignments by EMBOSS Needle at EMBOSS^43^ (https://www.ebi.ac.uk/jdispatcher/psa/emboss_needle) at the amino acid and DNA sequence level by comparing with reference sequences of CLCuMuV-Raj. The NCBI conserved domain database (CDD) and InterPro Scan at EMBL-EBI were employed for inferring the domains structure information^44^. PSIPRED was utilized to predict PSI-BLAST profile-based secondary structure prediction of reference and truncated sequence of C1 and C4 proteins^45^. Furthermore, the conserved functional motifs were predicted using MEM Motif Discovery tools. Moreover, the protein structures were investigated by subjecting the proteins sequences to DeepMind’s AlphaFold Protein Structure Database (AlphaFold DB)-AlphaFold2, which uses artificial intelligence and machine learning to give accurately predicted protein structure^46^. The protein structures were visualized and structurally aligned with mutated partner protein and root mean square deviation (RMSD) for quantitative measurement of the similarity between two superimposed atomic coordinates was executed by PyMOL^47^. The calmodulin-binding functional motif was predicted in C1 proteins using the Calmodulin Target database. The physicochemical parameters in C1 and C4 full-length and mutated proteins were detected through ExPASy ProtPram (https://web.expasy.org/protparam/).

### Plant material collection and use permission

For research experiments and collection of cotton plants, all relevant permits or permissions have been obtained from Biosafety Committee, National Institute for Biotechnology and Genetic Engineering. No voucher specimens are involved in this research work.

### Complies with international, national and/or institutional guidelines

The present experimental research and field studies on cotton comply with relevant institutional, national, and international guidelines and legislation.

## Results

### Cotton leaf curl disease (CLCuD) scenario in Pakistan

The CLCuD complex survey of 2020–2021 revealed the current situation of CLCuD, their evolution and spread from major cotton-growing areas of Punjab to Sindh, province of Pakistan. Since 2015, the CLCuD again appeared in the cotton-growing areas of central Punjab cities (Lahore, Faisalabad, Vehari, Burewala, Multan and DG Khan)^26^. Moreover, the prevalent strain of begomovirus-betasatellite CLCuMuV-Raj/CLCuMuB^Veh^ were reported while we identified the same complex from central to southern Punjab cities (Faisalabad, Lodhran, Bahawalpur, Rahimyar Khan) and neighboring cities, Tandojam which is in Sindh province, Pakistan. The details of each clone of begomovirus and satellites have been described in Tables 1 and 2, respectively.

**Table 1:** Genomic properties of CLCuD begomoviruses obtained from infected cotton samples.

Sr. no	Acc. no	Year	Virus	Clone	Size (nt)	Host	City	C1 (aa)	C2 (aa)	C3 (aa)	C4 (aa)	C5 (aa)	V1 (aa)	V2 (aa)
1	MW654014	2020	CLCuMuV-Raj	MAM-1	2738	Cotton	BWP	362 (2583–1495)	150 (1598–1146)	134 (1453–1049)	94 (2429–2145)	243 (791–60)	256 (276–1046)	118 (116–472)
2	MW654015	2020	CLCuMuV-Raj	MAM-2	2738	Cotton	BWP	362 (2583–1495)	150 (1598–1146)	134 (1453–1049)	94 (2429–2145)	243 (791–60)	256 (276–1046)	118 (116–472)
3	MW654016	2020	CLCuMuV-Raj	MAM-3	2738	Cotton	BWP	362 (2583–1495)	150 (1598–1146)	134 (1453–1049)	94 (2429–2145)	243 (791–60)	256 (276–1046)	118 (116–472)
4	MW654017	2020	CLCuMuV-Raj	MAM-4	2738	Cotton	BWP	362 (2583–1495)	150 (1598–1146)	134 (1453–1049)	94 (2429–2145)	243 (791–60)	256 (276–1046)	118 (116–472)
5	MW654018	2020	CLCuMuV-Raj	MAM-5	2738	Cotton	BWP	362 (2583–1495)	150 (1598–1146)	134 (1453–1049)	94 (2429–2145)	243 (791–60)	256 (276–1046)	118 (116–472)
6	MW654019	2020	CLCuMuV-Raj	MAM-6	2738	Cotton	LOD	362 (2583–1495)	150 (1598–1146)	134 (1453–1049)	94 (2429–2145)	243 (791–60)	256 (276–1046)	118 (116–472)
7	MW654020	2020	CLCuMuV-Raj	MAM-9	2738	Cotton	LOD	362 (2583–1495)	150 (1598–1146)	134 (1453–1049)	94 (2429–2145)	243 (791–60)	256 (276–1046)	118 (116–472)
8	MW654021	2020	CLCuMuV-Raj	MAM-10	2738	Cotton	LOD	362 (2583–1495)	150 (1598–1146)	134 (1453–1049)	94 (2429–2145)	243 (791–60)	256 (276–1046)	118 (116–472)
9	MW654022	2020	CLCuMuV-Raj	MAM-11	2738	Cotton	RYK	362 (2583–1495)	150 (1598–1146)	134 (1453–1049)	94 (2429–2145)	243 (791–60)	256 (276–1046)	118 (116–472)
10	MW654023	2020	CLCuMuV-Raj	MAM-12	2738	Cotton	RYK	362 (2583–1495)	150 (1598–1146)	134 (1453–1049)	94 (2429–2145)	243 (791–60)	256 (276–1046)	118 (116–472)
11	MW654024	2020	CLCuMuV-Raj	MAM-14	2738	Cotton	RYK	362 (2583–1495)	150 (1598–1146)	134 (1453–1049)	100 (2429–2127)	243 (791–60)	256 (276–1046)	118 (116–472)
12	MZ305187	2020	CLCuMuV-Raj	AV-40	2738	Cotton	TJ	362 (2583–1495)	150 (1598–1146)	134 (1453–1049)	100 (2429–2127)	243 (791–60)	256 (276–1046)	118 (116–472)
13	ON312776	2021	CLCuMuV-Raj	NIA-2	2738	Cotton	TJ	362 (2583–1495)	150 (1598–1146)	134 (1453–1049)	100 (2429–2127)	243 (791–60)	256 (276–1046)	118 (116–472)
14	ON312777	2021	CLCuMuV-Raj	NIA-5	2738	Cotton	TJ	362 (2583–1495)	150 (1598–1146)	134 (1453–1049)	100 (2429–2127)	243 (791–60)	256 (276–1046)	118 (116–472)
15	ON312778	2021	CLCuMuV-Raj	NIA-6	2738	Cotton	TJ	362 (2583–1495)	150 (1598–1146)	134 (1453–1049)	100 (2429–2127)	243 (791–60)	256 (276–1046)	118 (116–472)
16	ON312779	2021	CLCuMuV-Raj	NIA-8	2738	Cotton	TJ	362 (2583–1495)	150 (1598–1146)	134 (1453–1049)	100 (2429–2127)	243 (791–60)	256 (276–1046)	118 (116–472)
17	ON312780	2021	CLCuMuV-Raj	NIA-12	2738	Cotton	TJ	362 (2583–1495)	150 (1598–1146)	134 (1453–1049)	100 (2429–2127)	243 (791–60)	256 (276–1046)	118 (116–472)
18	ON312781	2021	CLCuMuV-Raj	S2-1	2740	Cotton	FSD	344 (2583–1549)	150 (1598–1146)	134 (1453–1049)	100 (2429–2127)	243 (791–60)	256 (276–1046)	118 (116–472)
19	ON312782	2021	CLCuMuV-Raj	S2-2	2740	Cotton	FSD	344 (2583–1549)	150 (1598–1146)	134 (1453–1049)	100 (2429–2127)	243 (791–60)	256 (276–1046)	118 (116–472)
20	ON312783	2021	CLCuMuV-Raj	S2-3	2740	Cotton	FSD	344 (2583–1549)	150 (1598–1146)	134 (1453–1049)	100 (2429–2127)	243 (791–60)	256 (276–1046)	118 (116–472)
21	ON312784	2021	CLCuMuV-Raj	S2-4	2740	Cotton	FSD	344 (2583–1549)	150 (1598–1146)	134 (1453–1049)	100 (2429–2127)	243 (791–60)	256 (276–1046)	118 (116–472)
22	ON312785	2021	CLCuMuV-Raj	S2-5	2740	Cotton	FSD	344 (2583–1549)	150 (1598–1146)	134 (1453–1049)	100 (2429–2127)	243 (791–60)	256 (276–1046)	118 (116–472)
23	ON312786	2021	CLCuMuV-Raj	S2-6	2740	Cotton	FSD	344 (2583–1549)	150 (1598–1146)	134 (1453–1049)	100 (2429–2127)	243 (791–60)	256 (276–1046)	118 (116–472)
24	ON312787	2021	CLCuMuV-Raj	S2-7	2740	Cotton	FSD	344 (2583–1549)	150 (1598–1146)	134 (1453–1049)	100 (2429–2127)	243 (791–60)	256 (276–1046)	118 (116–472)
25	ON312788	2021	CLCuMuV-Raj	S2-8	2740	Cotton	FSD	344 (2583–1549)	150 (1598–1146)	134 (1453–1049)	100 (2429–2127)	243 (791–60)	256 (276–1046)	118 (116–472)

*BWP* Bahawalpur, *LOD* Lodhran, *RYK* Rahimyar Khan, *TJ* Tandojam.

**Table 2:** Reading frames of alpha-satellites and beta-satellites identified in current study.

Sr. no	Acc. no	Year	Satellite name	Clone	Size (nt)	Host	City	βC1	Rep
1	MW654034	2020	CLCuMuA	MAM-15	1371	Cotton	BWP	–	315 (77–1024)
2	MW654035	2020	CLCuMuA	MAM-16	1371	Cotton	LOD	–	315 (77–1024)
3	MW654036	2020	OLCuA	MAM-18	1371	Okra	LOD	–	315 (77–1024)
4	MZ057237	2020	CLCuMuA	AV-16	1372	Cotton	TJ	–	315 (77–1024)
5	MZ057238	2020	CLCuMuA	AV-17	1372	Cotton	TJ	–	315 (77–1024)
6	MZ057239	2020	CLCuMuA	AV-18	1371	Cotton	TJ	–	315 (77–1024)
7	MZ057240	2020	CLCuMuA	AV-19	1372	Cotton	TJ	–	315 (77–1024)
8	MZ057241	2020	CLCuMuA	AV-21	1370	Cotton	RYK	–	315 (77–1024)
9	MZ057242	2020	CLCuMuA	AV-22	1371	Cotton	RYK	–	315 (77–1024)
10	MZ057243	2020	CLCuMuA	AV-23	1399	Cotton	RYK	–	132 (truncated)
11	MZ057244	2020	CLCuMuA	AV-26	1370	Cotton	MUL	–	315 (77–1024)
12	MZ057245	2020	CLCuMuA	AV-27	1371	Cotton	MUL	–	315 (77–1024)
13	MZ057246	2020	CLCuMuA	AV-28	1370	Cotton	MUL	–	315 (77–1024)
14	MZ057247	2020	CLCuMuA	AV-29	1370	Cotton	VEH	–	315 (77–1024)
15	MZ057248	2020	CLCuMuA	AV-30	1370	Cotton	VEH	–	315 (77–1024)
16	MZ057249	2020	OLCuA	AV-31	1370	Okra	VEH	–	315 (82–1029)
17	MZ057250	2020	OLCuA	AV-32	1370	Okra	VEH	–	315 (82–1029)
18	MZ057251	2020	CLCuMuA	AV-34	1370	Cotton	VEH	–	315 (77–1024)
19	MZ057252	2020	OLCuA	AV-35	1370	Okra	VEH	–	315 (82–1029)
20	ON312789	2021	CLCuMuA	V1-4	1372	Cotton	FSD	–	315 (77–1024)
21	ON312790	2021	CLCuMuA	V1-5	1372	Cotton	FSD	–	315 (77–1024)
22	ON312791	2021	CLCuMuA	V2-2	1370	Cotton	FSD	–	315 (77–1024)
23	ON312792	2021	CLCuMuA	V2-3	1370	Cotton	FSD	–	315 (77–1024)
24	ON312793	2021	CLCuMuA	V2-7	1370	Cotton	FSD	–	315 (77–1024)
25	ON312794	2021	CLCuMuA	S2-3	1371	Cotton	FSD	–	315 (77–1024)
26	ON312795	2021	CLCuMuA	S1-2	1371	Cotton	FSD	–	315 (77–1024)
27	ON312796	2021	CLCuMuA	S1-3	1371	Cotton	FSD	–	315 (77–1024)
28	ON312797	2021	GDarSLA	S1-1	1364	Cotton	FSD	–	315 (70–1017)
29	ON312798	2021	GDarSLA	S2-1	1364	Cotton	FSD	–	315 (70–1017)
30	ON312799	2021	GDarSLA	S2-2	1364	Cotton	FSD	–	315 (70–1017)
31	ON312800	2021	GDarSLA	S2-4	1364	Cotton	FSD	–	315 (70–1017)
32	ON312801	2021	OLCuA	V1-1	1375	Cotton	FSD	–	315 (82–1029)
33	ON312802	2021	OLCuA	V1-2	1376	Cotton	FSD	–	315 (82–1029)
34	ON312803	2021	OLCuA	V1-3	1375	Cotton	FSD	–	315 (82–1029)
35	MW654025	2020	CLCuMuB	MAM-22	1359	Cotton	BWP	118 (550–194)	–
36	MW654026	2020	CLCuMuB	MAM-24	1359	Cotton	BWP	118 (550–194)	–
37	MW654027	2020	CLCuMuB	MAM-25	1359	Cotton	RYK	118 (550–194)	–
38	MW654028	2020	CLCuMuB	MAM-28	1359	Cotton	RYK	118 (550–194)	–
39	MW654029	2020	CLCuMuB	MAM-29	1359	Cotton	RYK	118 (550–194)	–
40	MW654030	2020	CLCuMuB	MAM-31	1359	Cotton	LOD	118 (550–194)	–
41	MW654031	2020	CLCuMuB	MAM-33	1359	Cotton	LOD	118 (550–194)	–
42	MW654032	2020	CLCuMuB	MAM-34	1359	Cotton	TJ	118 (550–194)	–
43	MW654033	2020	CLCuMuB	MAM-35	1359	Cotton	TJ	118 (550–194)	–
44	ON312804	2021	CLCuMuB	NIA-3	1358	Cotton	TJ	118 (550–194)	–
45	ON312805	2021	CLCuMuB	NIA-4	1269	Cotton	TJ	118 (551–195)	–
46	ON312806	2021	CLCuMuB	V1	1370	Cotton	TJ	118 (551–195)	–
47	ON312807	2021	CLCuMuB	V2	1358	Cotton	TJ	118 (551–195)	–

*MUL* Multan, *VEH* Vehari, *FSD* Faisalabad, *TJ* Tandojam.

### BLASTn, ORF, and SDT analysis of begomovirus and alphasatellites

**Figure 2:**
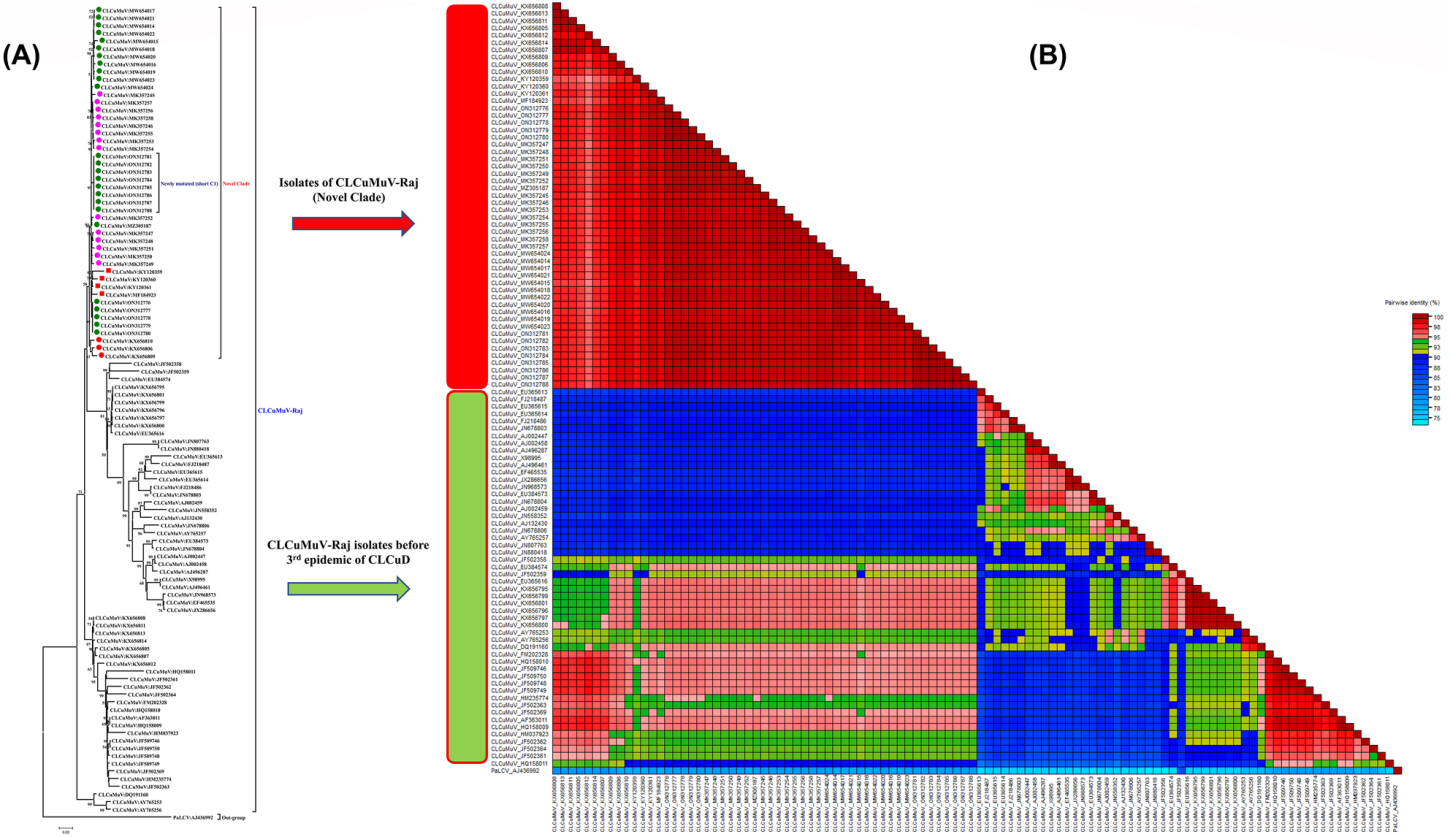
Phylogenetic relationship of present CLCuD-begomoviruses with other begomoviruses available in the NCBI-GenBank. (**A**) A neighbor-joining phylogenetic tree was reconstructed to infer the relationship of the isolates found in the current study. The tree was developed using the different sequences of CLCuMuV (including the reference sequences of each strain of CLCuMuV). The multiple sequence alignment was carried out by MUSCLE program in MEGA7 software ^40^. The tree was developed with 1000 bootstrap value represented along each root. All the sequences identified in the current study belonged to the novel clade in CLCuMuV-Raj strain. Green dotted sequences were determined in the present study while purple and red colored sequences were retrieved from NCBI GenBank, showed CLCuD-begomoviruses sequences collected from the survey from Pakistan and India, respectively. (**B**) DNA-A molecules were aligned, and identity percentage were calculated using SDT program. The Papaya leaf curl virus (PaLCV: AJ436992) was taken as an outgroup for the analysis.

A total of twenty-five begomovirus sequences were determined that were approximately 2.7 kb in size and composed of seven open reading (ORFs) frames. The details of the sample origin, ORFs and their accession numbers are shown in Table 1. All the begomovirus sequences identified in this study showed ~ 99–100% nucleotide sequence similarity among themselves. The cut-off value for sequence demarcation described by ICTV tells that ≥ 91% nucleotide identity shows that they belong to a single virus species, i.e., CLCuMuV. Their similarity results showed more than 99% sequence similarity with the recently reported isolates of the CLCuMuV-Raj strain from Pakistan in 2018^27^ (Fig. 2A).

Among 25 begomoviruses, 8 samples were collected from different fields of Tandojam in 2021 survey, were identified as CLCuMuV-Raj from where Luqman et al.^48^ identified the CLCuKoV and CLCuShV from the main cotton growing areas of Sindh in 2010. SDT analysis showed more than 99% sequence identity scores of the current isolates with other sequences of the CLCuMuV-Raj strain (all belonging to the novel clade in phylogenetic analysis; Fig. 2B). Whereas, among 17 begomoviruses identified from the southern Punjab and Sindh province cities (Bahawalpur, Lodhran, Rahimyar Khan, and Tandojam) in a 2020 survey while 10 samples (MW654014–MW654023) which were from southern Punjab cities (Bahawalpur, Lodhran and Rahimyar Khan) have 94 aa C4 protein instead of 100 aa in a result of early stop codon.

**Figure 3:**
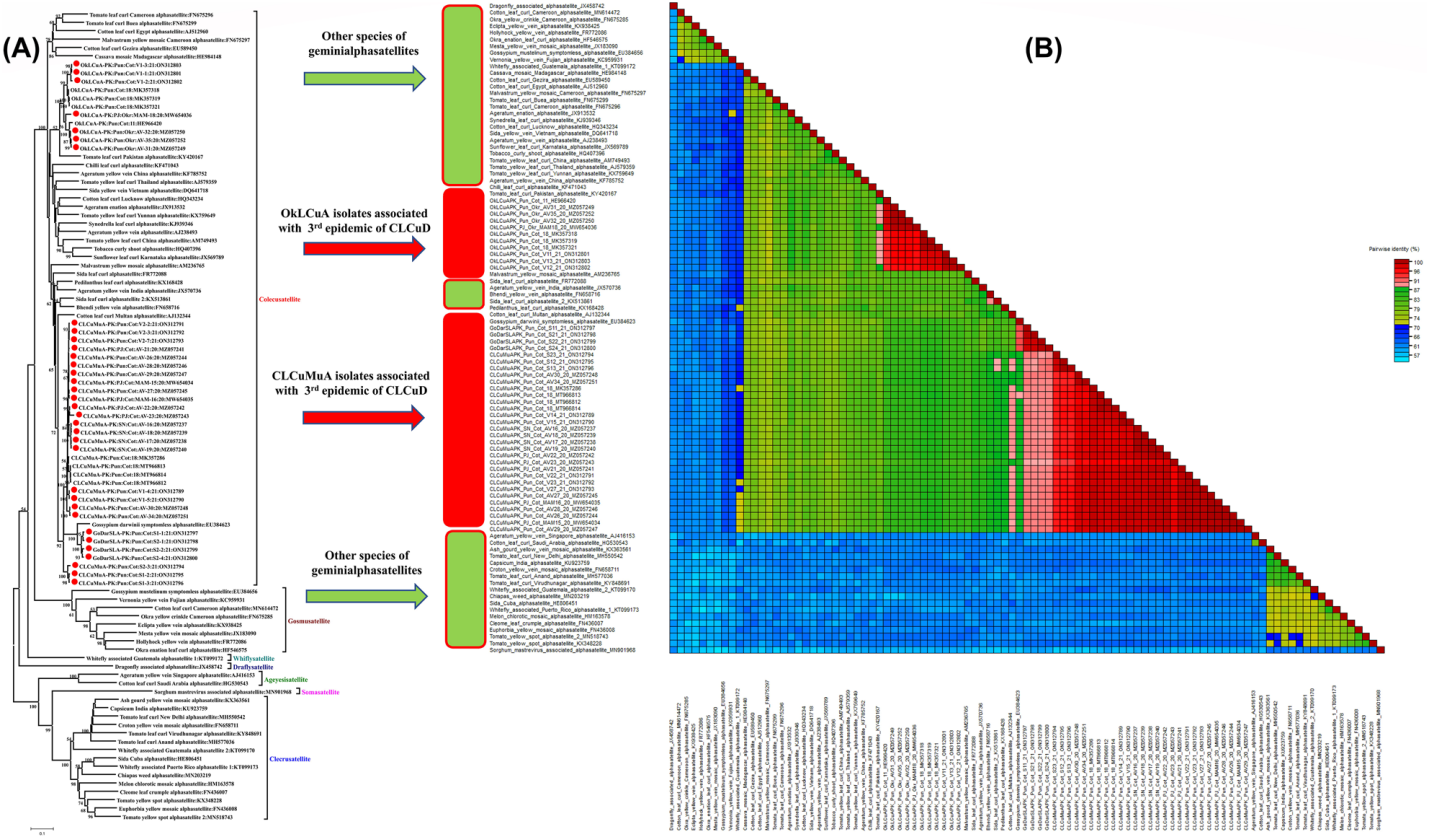
Bayesian phylogenetic and SDT analysis alphasatellites (CLCuMuA). (**A**) A neighbor-joining phylogenetic tree was reconstructed to infer the relationship of the isolates of alphasatellites found in the current study. The tree was developed using the different sequences of reference species of alphasatellites plus a few sequences identified in similarity searches of the current sequences. The multiple sequences alignment was carried out by MUSCLE program in MEGA7 software^40^. The bootstrap method (with 1000 replications) was used. 16 sequences identified in the current study belonged to the CLCuMuA and others belonged to OkLCuA. The isolates of the current study are labelled with red circles. (**B**) The alphasatellite sequences were aligned and identity percentage were calculated using SDT program.

A total 34 sequences of alphasatellites were isolated in this study and the sequence accession numbers are mentioned in Table 2. These alphasatellites share ≥ 95% sequence identity (Fig. 3A) with the recently reported sequences of CLCuMuA from Pakistan in 2018^27^. They vary in size from 1370 to 1372 nucleotides. All the sequences had a typical Rep gene of ~ 945 nucleotides in the virion strand (Table 2). The SDT analysis showed more than 95% sequence identity scores of the current isolates with other sequences of CLCuMuA (Fig. 3B). This shows the stability of this strain in the field of cotton still in 2020. The ORFs of all the satellite molecules are shown in Table 2.

### Phylogenetic analysis of begomovirus and alphasatellite sequences

In neighborhood phylogenetic analysis, the tree was reconstructed with reference sequences along with the present begomovirus sequences (accession No. MW654014 to MW654024, and MZ305187). The sequences of the begomoviruses of the present study have made the phylogenetic clade with the recently reported sequences of the CLCuMuV-Raj strain (Fig. 2A). The novel clade was first time named in 2015 when a few sequences from India ^25^ made a novel clade with three sequences previously reported from Pakistan (KX656806, KX656809 and KX656810)^22^. Later, a few other sequences were submitted from India and fell into this novel clade.

In phylogenetic tree analysis, the sequences of the alphasatellites (MW654034, MW654035, MZ057237, MZ057238, MZ057239, MZ057240, MZ057241, MZ057242, MZ057243, MZ057244, MZ057245, MZ057246, MZ057247, MZ057248, and MZ057251) identified from cotton developed the clade with the recently, reported sequences of the alphasatellites and the reference sequences of the respective species (Fig. 3A).

### A single betasatellite species, CLCuMuB associated with CLCuD

**Figure 4:**
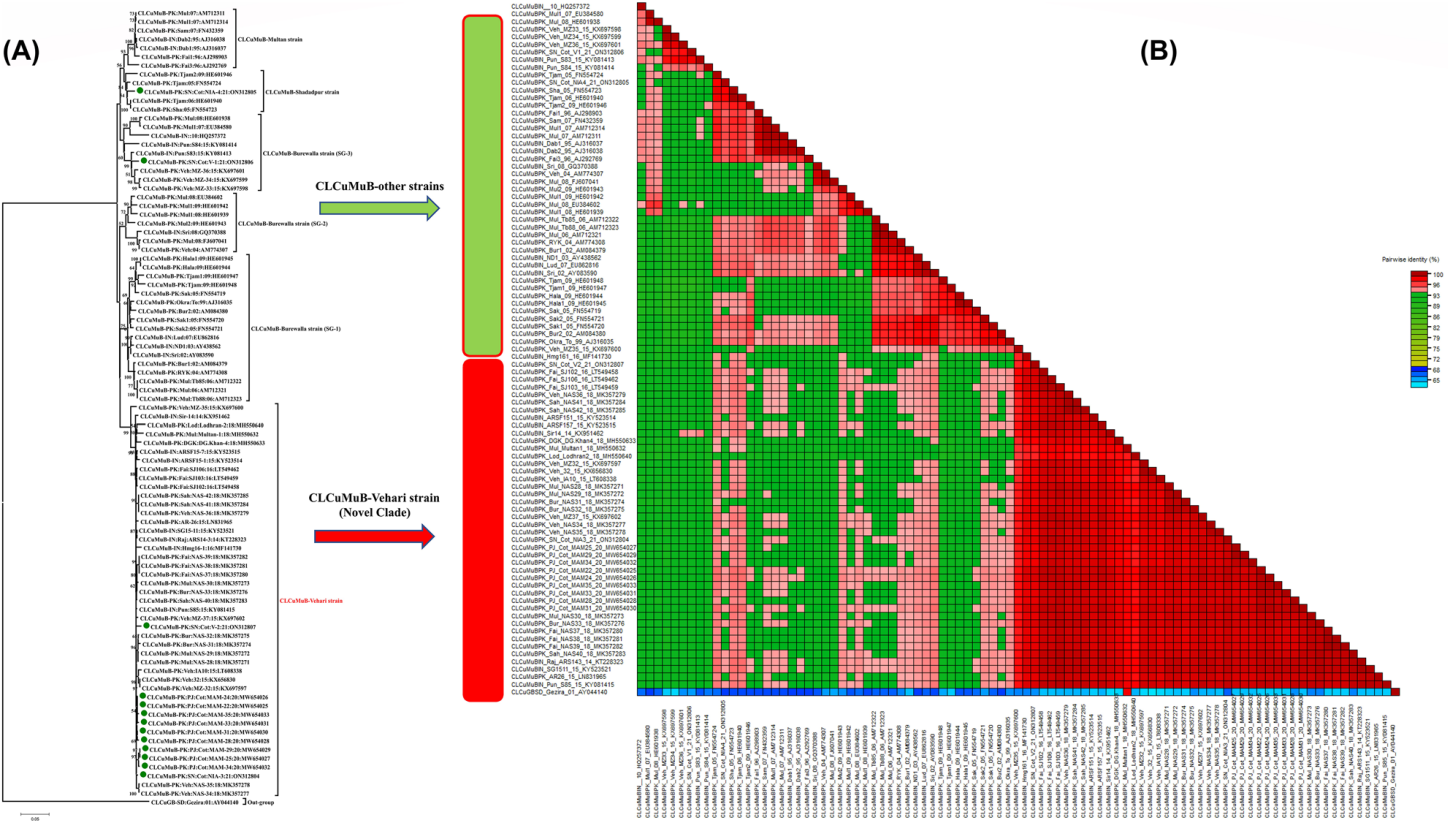
Bayesian phylogenetic and SDT analysis betasatellites (CLCuMuB). (**A**) A neighbor-joining phylogenetic tree was reconstructed to infer the relationship of the isolates of betasatellites found in the current study. The tree was developed using the different sequences of CLCuMuB. The bootstrap method (with 1000 replications) was used. All the sequences identified in the current study belonged to the CLCuMuB^Veh^ (Vehari strain). The isolates of the current study are labelled with green circles. (**B**) The betasatellite sequences were also aligned by MUSCLW integrated in SDT and identity percentage were calculated using SDT program. Cotton leaf curl Gezira betasatellite (CLCuGB; AY044140) was chosen as an outgroup for the analysis.

A total 13 betasatellite sequences were isolated in this study and their accession numbers are shown in Table 2. Nucleotide BLAST results of 13 betasatellite sequences (accession No. MW654025, MW654026, MW654027, MW654028, MW654029, MW654030, MW654031, MW654032, and MW654033) showed ≥ 95% identity to different CLCuMuB^Veh^ (Vehari strain) isolates that were identified in 2018 from cotton fields of Pakistan. All the betasatellite sequences were the same in length (1359 nucleotides) and had a ~ 354 nucleotide βC1 gene in the complementary strand. The betasatellite (CLCuMuB) sequences found in this current study had 99% nucleotide identity among themselves (Fig. 4A). Based on the sequence demarcation cut-off value at ≥ 91% nucleotide identity^37^, the isolated betasatellites are the members of CLCuMuB that reveal the occurrence of single betasatellite species with the outbreak of CLCuD in Pakistan. The ORFs of all the satellite molecules are shown in Table 2. Recently Zubair et al.^22^ reported three different types of betasatellites including CLCuMB^Bur^, CLCuMB^Veh^, and CLCuMB^Mul^ in association with CLCuD-begomovirus complex in Pakistan. The SDT analysis of betasatellites identified in the current study showed more than 99% sequence identity scores of the current isolates with other sequences of the CLCuMuB^Veh^ strain (all belonging to the novel clade in the above phylogenetic analysis; Fig. 4B).

In the phylogenetic tree analysis, the sequences of the betasatellites developed the clade with the recently reported sequences of the CLCuMuB^Veh^ strain that had made the distinct clade in CLCuMuB. This distinct clade consists of the isolates of CLCuMuB identified after 2014 from Pakistan and India. In 2018, in a broad survey of Pakistan, it was realized that CLCuMuB^Veh^ became dominant in the field. The tree was rooted on Cotton leaf curl Gezira betasatellite (CLCuGB; AY044140) as an outgroup. This shows the stability of the CLCuMuB^Veh^ in the field.

### Mutation and protein modeling of C1 and C4

**Figure 5:**
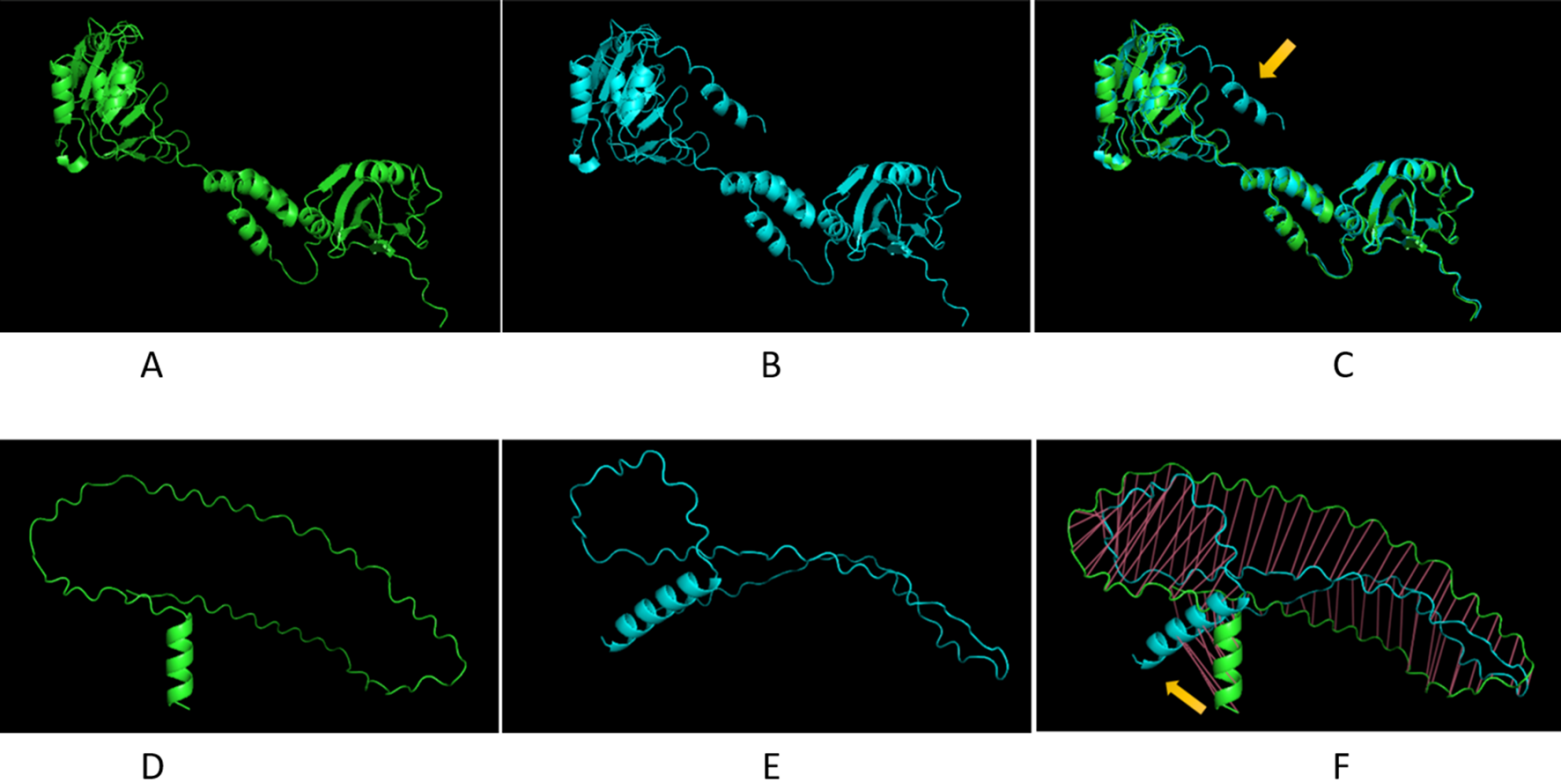
Protein structure prediction and alignment. DeepMind’s AlphaFold predicted structures of (**A**) C1 mutated protein (**B**) C1 protein identified in this study. (**C**) Protein structures were visualized, structurally aligned with mutated partner protein with 0.470 Å RMSD score, AlphaFold predicted structures of (**D**) C4 mutated protein (**E**) C4 protein identified in this study. (**F**) Protein structures were visualized, structurally aligned with mutated partner protein with 13.810 Å RMSD score the yellow arrow represents the left portion of full-length protein from superimposition.

We found 10 C4 mutated CLCuMuV-Raj begomoviruses from the BWP, LOD, and RYK in the year of 2020 while two begomoviruses from RYK and TJ have complete *C4 gene*, exhibited the spread of mutation toward Sindh province of Pakistan. Then, in the survey of 2021 from central Punjab (FSD) and Sindh (TJ) provinces showed the C1 mutation in eight sequences (ON312781- ON312788) identified from the different fields of FSD. However, we found full *C1* and *C4* genes of CLCuMuV-Raj begomoviruses identified from Sindh which exhibits that mutated genes still may not be prevalent in Sindh province. The sequence level comparison of the identified mutant, and reference viral genome demonstrated a significant mutation in DNA fragment at the N-terminal of *C1* and *C4* genes (Fig. 5A–F). These single nucleotide polymorphisms (SNPs) converted a functional codon L (leucine amino acid in C1) and Q (Glutamic in *C4* gene) into stop codon causing truncation of C1 (362 aa) into C1 (344 aa) and C4 (100 aa) into C4 (94 aa) (Figs. S1 and S2). The mutated *C1* (344 aa) was fully matched with full-length *C1* (362 aa) gene and a complete Gemini_AL1 conserved domain was observed (Fig. S1). Similarly, the mutated C4 protein (94 aa) was fully aligned with the complete C4 protein (100 aa) except for the last N-terminal six amino acids (deletion fragment) (Fig. S2).

The secondary structure assessment of mutant and reference also exhibited a significant difference e.g., C1 (362 aa) proteins exhibited extra coil and helix region (Fig. S3). Similarly, the C4 (100 aa) also showed one extra coil and six helixes as compared to C4 (94 aa) (Fig. S4). The 3D protein structures and their super imposition between mutant and reference displayed 0.470 Å RMSD score and 13.10 Å RMSD score in the mutant and normal proteins of C1 and C4, respectively. The multiple sequence alignment and MEME motif analysis also correspond to the above finding e.g., we observed the deletion of one conserved motif at the C-terminal of proteins. The calmodulin binding sites prediction between C1 (362 aa) and C1 (344 aa) was similar, showing conserveness of the main calmodulin binding activity of the current mutant virus in the spreading epidemic (Fig. S5). Similarly, the calmodulin-binding sites were found conserved in C4 (100 aa) and C4 (94 aa) (Fig. S6). Moreover, we identified the physicochemical parameters in C1 and C4 full-length and mutated proteins which show certain differences in truncated and full-length proteins (Table 3). Based on in silico protein structure and physicochemical properties prediction, it is deduced that truncated C1 and C4 are different from the full-length versions and might be associated with the 3rd epidemic. Though, wet lab biochemical studies will be further required to provide more evidence of these predictions, yet these are beyond the scope of present study.

**Table 3:** Physicochemical parameters predictions in C1 and C4 full length and truncated proteins.

Physicochemical parameters	C1 full length	C1 truncated	C4 full length	C4 truncated
Number of amino acids	362	344	100	94
Molecular weight	40,957.88	38,994.96	10,988	10,275.32
Theoretical pI	6.28	7.17	10.05	10.06
Atomic composition				
Carbon (C)	1828	1753	459	429
Hydrogen (H)	2796	2677	740	688
Nitrogen (N)	502	477	148	138
Oxygen (O)	554	517	156	146
Sulfur (S)	9	9	5	5
Total number of atoms	5689	5433	1508	1406
Aliphatic index	69.56	72.62	49.8	44.68
Grand average of hydropathicity	− 0.644	− 0.572	− 0.853	− 0.857

## Discussion

**Figure 6:**
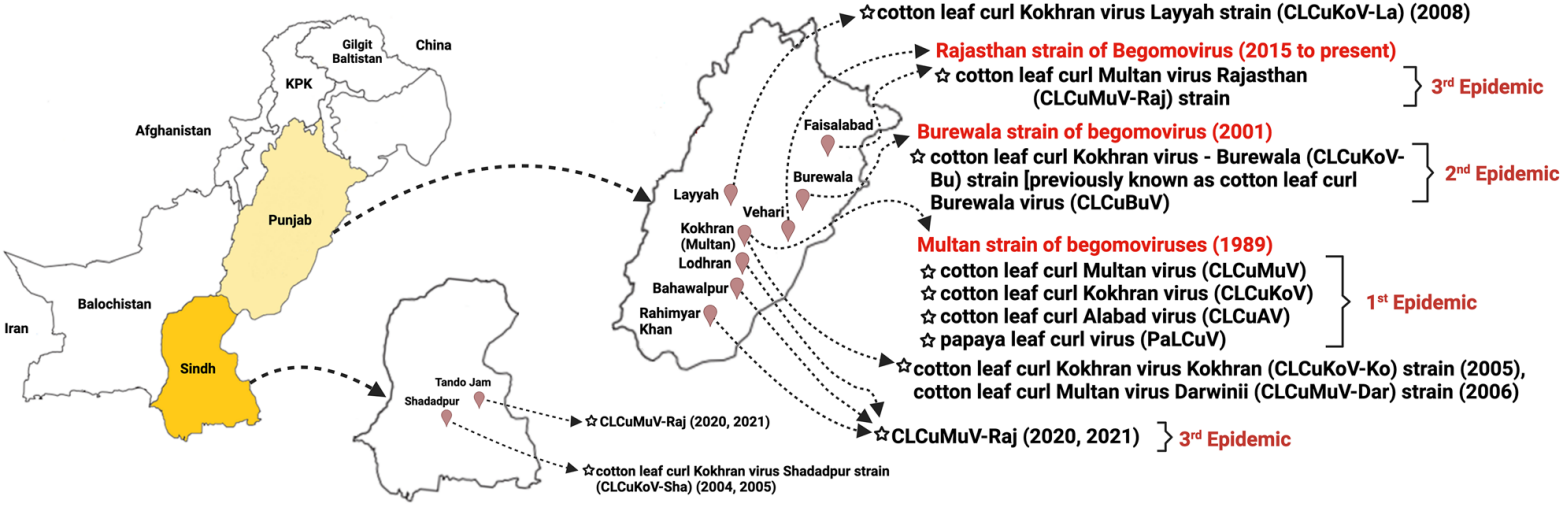
Geographic distribution of CLCuD in Pakistan. Graphical illustration exhibited the emergence of CLCuD with respect to cities. Adapted from Mahmood et al.^58^.

Cotton, like other countries, remains an economically and commercially important crop for Pakistan. Two provinces, Sindh and Punjab have been recorded as the top cotton production areas of Pakistan but at the same time, these are vulnerable to CLCuD and pink bollworm (*Pectinophora gossypiella*), resulting in severe crop losses every year. In the Punjab province, CLCuD has passed through three major epidemics during the past three decades (Fig. 6). During the first epidemic of CLCuD in Pakistan and north-western India, distinct begomovirus species (CLCuMuV, CLCuAlV, CLCuKoV, PaLCuV, CLCuRaV, and ToLCBaV) and associated satellites (CLCuMuB and CLCuMuA) were identified from cotton. Moreover, another species Cotton leaf curl Bangalore virus (CLCuBaV) associated with CLCuD was identified from Southern India^49^. CLCuBaV was associated with *Kenaf* leaf curl betasatellite (KeLCuB) which was identified from malvaceous fibre crop kenaf (*Hibiscus canabinus*). However, CLCuBaV and KeLCuB were not identified from the Indian subcontinent CLCuD epidemic. The disease disappeared with the adoption of resistant varieties, but virus continued its emergence and evolution to produce more virulent strains of begomoviruses that resulted in the resistance breakdown in 2001. This resistance breakdown was associated with the single begomovirus, CLCuKoV Burewala strain (CLCuKoV-Bur) which was reported in Burewala, Vehari district, Punjab, Pakistan. The virus was associated with a recombinant betasatellite CLCuMuB^Bur^, smudging the subcontinent with second CLCuD epidemic^50,51^. Although the virus was not reported from Sindh province at that time, CLCuD has become a problem there since 2004, although not on the scale of the rest of the country^52^. Recent studies of the viruses have exhibited a more diversity of begomoviruses in Punjab than north such as CLCuKoV (i.e., part of the first epidemic in the 1990s) Cotton leaf curl Gezira virus (CLCuGeV) and CLCuShV. The CLCuBuV and CLCuShV have recombinant genomes derived from CLCuMuV and CLCuKoV. The situation of CLCuD in India was different from Pakistan, since in 1994, CLCuD was first reported near Rajasthan state (Sri Ganganagar) adjoining Pakistan which spread into the Punjab and rest of the north region of the Indian cotton growing zone within 4–5 years^53–55^. The dominant strain of begomovirus, CLCuMuV-Raj was reported in those years but the drastic changed have been noticed in 2004–2005, when the resistance-breaking strain, CLCuKoV-Bur was identified in the northwestern India which was further remained dominant till 2009–2010 and has been the cause of the severe outbreak of CLCuD in Punjab and Rajasthan states of India. Since then, CLCuKoV-Bu has remained the single dominant cotton-associated begomovirus in India and Pakistan^50,56,57^.

The CLCuKoV-Bur strain was dominant till 2014 but the situation changed in 2015 when CLCuMuV bounced back in cotton both in Pakistan and India^22,58^. In 2015–2016, two independent studies were conducted on CLCuD in India and Pakistan by Zubair et al.^50^ and Datta et al.^25^. Zubair et al.^50^ investigated the CLCuD complex by collecting samples from cotton fields at Vehari and performed illumine sequencing. They found three distinct begomovirus species (CLCuKoV, CLCuAlV, and CLCuMuV) with full *TrAP* associated with distinct species of alphasatellites and betasatellites. Furthermore, they also found a new strain of betasatellites i.e., CLCuMuB^Veh^. During 2015–2016, in southern Punjab India, a massive attack of whiteflies on cotton fields led to heavy CLCuD infestation that caused complete destruction of 2/3rd area covering 1.15 million acres of grown cotton. They did not find CLCuKoV-Bu from the fields and identified a distinct clade of CLCuMuV and also reported the prediction of a third epidemic of CLCuD in the Indian subcontinent^25^. Another study from India surveyed cotton growing areas of Northwestern zone and covered three different states adjoining the Punjab, Pakistan. They conducted sampling from Haryana state (Fatehabad, Rohtak, Hisar, Sirsa); Punjab state (Bathinda, Faridkot, Fazilka and Mansa) and Rajasthan state (Hanumangarh and Sri Ganganagar) in 2012–2014 and found four distinct begomovirus strains [CLCuMuV-Raj, CLCuMuV-Pakistan (PK), CLCuMuV-Faisalabad (Fai) and CLCuKoV-Bu]. However, CLCuMuV-Raj was the most prevalent begomovirus in northwestern zone of India which is adjoining to the Punjab province of Pakistan^59^. Another recent study by Iqbal et al. performed a sentinel experiment from 2012 to 2017 to check the virus complex from cotton growing areas of upper to central Punjab (Lahore, Multan, Vehari) of Pakistan and performed population analysis experiments, resulting in CLCuMuV-Raj expansion displacement of CLCuKoV-Bu from both in India and Pakistan^60^.

Afterward, based on statistical data from begomovirus diversity and climatic changes, a third CLCuD epidemic was predicted in the Indian subcontinent after the year 2016^25,56^. This prediction was supported by our recent study on field data and hotspot analysis of CLCuD in Punjab, Pakistan^27^, where some hotspots were identified in data of 2014–2018; however, the CLCuD indices were documented as 24% and 29% for 2017 and 2018, respectively. The disease hotspots were found in major cotton-growing areas of Punjab, from central Punjab to upper Punjab up to the Multan region which showed CLCuMuV-Raj dominance in the Punjab, Pakistan^27^.

In the present study, we have exclusively identified the genetic diversity allied with the epidemic in the regions ahead of Multan, mostly called Southern Punjab. The prime reason for the usage of universal primers is to explore whether prevalent strain (CLCuMuV-Raj) is still dominant in the cotton fields, or it may spread to the southern part of Pakistan. Furthermore, several studies have been used these universal primers along with RCA restriction-based cloning^22,27^ and were not found any difference. Moreover, we also have expanded the picture by sampling from the border regions of the Punjab-Sindh and Sindh areas. We identified the CLCuMuV-Raj strain as predominant in all the study area locations which shows the stability of the CLCuMuV-Raj strain not only in the region of Punjab but also indicates its spread in Sindh, where previously CLCuKoV and CLCuShV were reported from cotton areas^48^. Biswas et al. showed similar findings in the Northwestern areas of Punjab, India. The NW Punjab, India is geographically linked to the Punjab and Sindh, Pakistan, and shares similar climate conditions. These findings are concurrent with the previous and present studies from Pakistan and India showing the time-to-time paradigm shift of CLCuD-associated begomovirus, most importantly signifying the return of CLCuMuV-Raj in Punjab and Sindh of Pakistan and India after 2016^25,59,61^.

DNA viruses associated with CLCuD exhibited extreme genomic plasticity, resulting in enhanced virulence and rapid evolution under different cropping systems along with an extension in host range. Full-length CLCuMuV-Raj begomovirus found from the samples of Punjab (BWP, LOD, RYK) showed a mutation in C4 protein which provide the evidence of rapid recombination events. Moreover, the size is reduced to 94 amino acids (aa) as compared to the previously identified isolates from seven central Punjab cities (FSD, Toba Tek Singh, Khanewal, Sahiwal, Multan, Vehari, Burewala) where complete C4 (100 aa) have been found^27^. The in-silico prediction of both 100 aa and 94 aa C4 proteins also showed the presence of less helix and coiled region in mutated protein. The C4 protein in geminiviruses helps in efficient viral infection and development of disease symptoms^61^. It also interacts with different host plant genes to suppress plant transcriptional and post-transcriptional gene silencing^62^. Mutation in the C4 gene in CLCuKoV show the reduced symptoms development yet this mutation did not affect the maintenance of associated betasatellite^63^. Moreover, a mutant of malvastrum yellow vein virus (MaYVV) with defective C4 protein, in association with its betasatellite showed enhanced levels of viral accumulation and infection in tobacco^64^. The mutation of the C4 protein in our study might pinpoint its towards successful viral infection in the shifting scenario of begomoviruses associated with the third pandemic of CLCuD. In addition to C4 mutation in CLCuMuV-Raj begomovirus, we also found 8 C1 mutated CLCuMuV-Raj in 2021 survey of cotton fields from central Punjab but we did find any C1 or C4 mutation in any CLCuMuV-Raj sequences from cotton growing areas of Sindh (TJ).

Further, we identified a single predominant betasatellite associated with the disease i.e., CLCuMuB which is also similar to the previous findings^27^. Zubair et al. reported three different types of betasatellites including CLCuMB^Bur^, CLCuMB^Veh^, and CLCuMB^Mul^ in association with CLCuD-begomovirus complex in Pakistan till the year 2015^22^. Ahmed et al. showed the predominance of CLCuMB^Veh^ linked to the third epidemic^27^. Our findings exhibit the same trend and demonstrate the predominance of the CLCuMB^Veh^ strain associated with the CLCuD. We detected CLCuMuA alphasatellite as predominant in Punjab and Sindh areas, which is consistent with the previous study^25,27^. From the same fields, we further collected okra samples that were showing severe curling and yellowing. We isolated alpha satellites from infected okra samples also and identified OkLCA satellite from all those samples to check the diversity of alphasatellite as done by Ahmed et al.^27^ and Zubair et al.^22^ yet we only identified CLCuMuA highly dominant in cotton.

Whitefly transmitting the CLCuVs is of great importance in terms of disease spread and efficient transmission of a particular begomovirus complex. Better understanding of whitefly species helps in its sustainable mitigating. Several cultural, chemical, and biotechnological application have been employed to suppress the whitefly population from the field^65,66^. Kranthi et al. identified that Asia II 1 species of whitefly was found in the northwestern zone of India^67^. They collected whiteflies from infected cotton fields of three different zones (north, central and south) and found the prevalence of Asia II 1 in the north zone adjoining to central and southern Punjab province of Pakistan^67^. Furthermore, we recently reported the dominant occurrence of Asia II 1 in Punjab and Sindh, with respect to the third epidemic of CLCuD^68^. It is also determined that Asia II 1 efficiently transmits the CLCuMuV as compared to MEAM1 which on the contrary transmits TYLCV efficiently^69^. Another study presented the CLCuMuV-Raj identified from whitefly population cumulated from different cotton cultivation areas of Punjab^70^. Similar findings in our studies cognate with the available information and shed light on the dominance of whitefly Asia II 1 as successful transmitter of CLCuMuV-Raj in the current third CLCuD epidemic.

## Conclusions

The findings of this study elucidate the CLCuD complex linked with the present third epidemic in Pakistan. It adds information to previously carried out studies and covers the southern Punjab, Punjab-Sindh border, and major Sindh cotton-growing areas. Furthermore, to best of our knowledge, this is the first study exhibited the predominance of CLCuMuV-Raj strain associated with CLCuMB^Veh^ strain and its spread from southern Punjab province to Sindh province of Pakistan. The mutations identified in the *C4* and *C1* genes of begomoviruses are interesting and can be further explored in explaining the host-virus interaction in the third epidemic scenario. This study shows the current situation of CLCuD complex in the major cotton crop producing areas of Pakistan; it will help in understanding the re-emergence of CLCuD complex and timely management of economic damages caused by these viruses in the future.

### Supplementary Information

Supplementary Figures.

## Data Availability

The sequences generated during the current study were deposited in GenBank at NCBI with the accession numbers (MW654014-MW654024, MZ305187, ON312776-ON312788, MW654034-MW654036, MZ057237-MZ057252, ON312789-ON312703, MW654025-MW654033, ON312804-ON312807) given in Tables 1 and 2. These data is available online through NCBI repository.
