# Supplementary material for: Dominance of Cotton leaf curl Multan virus-Rajasthan strain associated with third epidemic of cotton leaf curl disease in Pakistan
Source: Sci Rep. 2024 Jun 12;14:13532. doi: 10.1038/s41598-024-63211-8 (PMC11169534; doi:10.1038/s41598-024-63211-8)
Supplement: Supplementary file 1 — Supplementary Figures. [file 41598_2024_63211_MOESM1_ESM.docx]

**Dominance of Cotton leaf curl Multan virus-Rajasthan strain associated with third epidemic of cotton leaf curl disease in Pakistan**

**Muhammad Arslan Mahmood^1†^, Nasim Ahmed^1,2^, Athar Hussain^1,3^, Rubab Zahra Naqvi^1^, Imran Amin^1^ and Shahid Mansoor^1,4^***

^1^Agricultural Biotechnology Division, National Institute for Biotechnology and Genetic Engineering (NIBGE) college Pakistan Institute of Engineering and Applied Sciences, Faisalabad, Pakistan

^2^Biotechnology and Microbiology Group, Department of Zoology, University of Poonch Rawalakot, Azad Jammu and Kashmir, Rawalakot, Pakistan

^3^Genomics Lab, School of Food and Agricultural Sciences (SFAS), University of Management and Technology (UMT), Lahore, 54000, Pakistan

^4^International Center for Chemical and Biological Sciences, University of Karachi, Karachi, Pakistan

**^†^**Current address: Plant Sciences Division, Research School of Biology, The Australian National University, Canberra, ACT 2601, Australia

***Correspondence: Shahid Mansoor:** [shahidmansoor7@gmail.com](mailto:shahidmansoor7@gmail.com)

**Protein alignment and domain analysis of C1 full-length and C1 mutated protein**


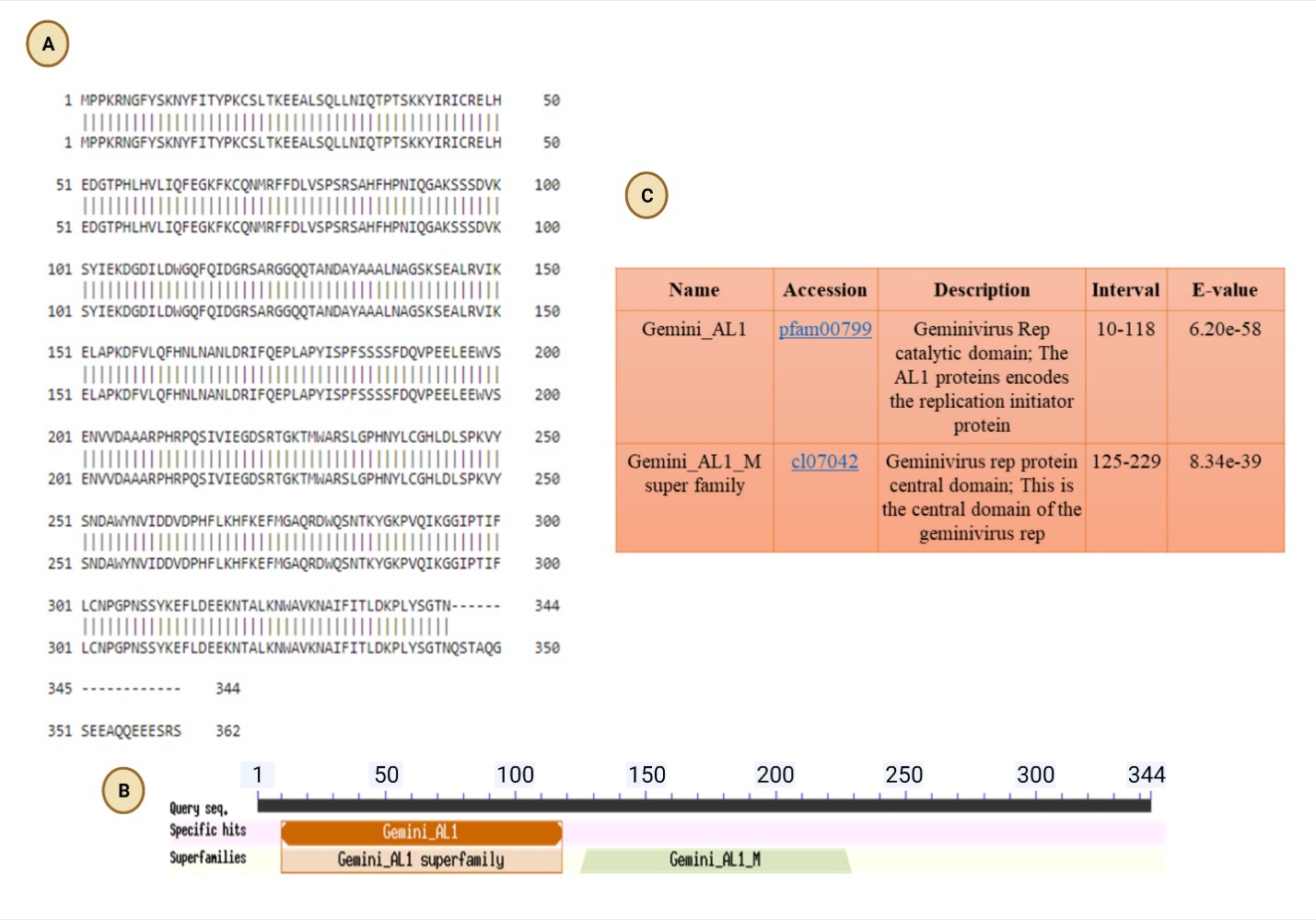


**Fig. S1.** (A) Alignment of C1 full-length protein (362 aa) with C1 mutated protein (344 aa) showed only 18 amino acids with gaps. (B & C) Domains of both versions of C1 proteins show the same Gemini_AL1 and Gemini_AL1_M superfamily.

**Protein alignment and domain analysis of C1 full-length and C1 mutated protein**


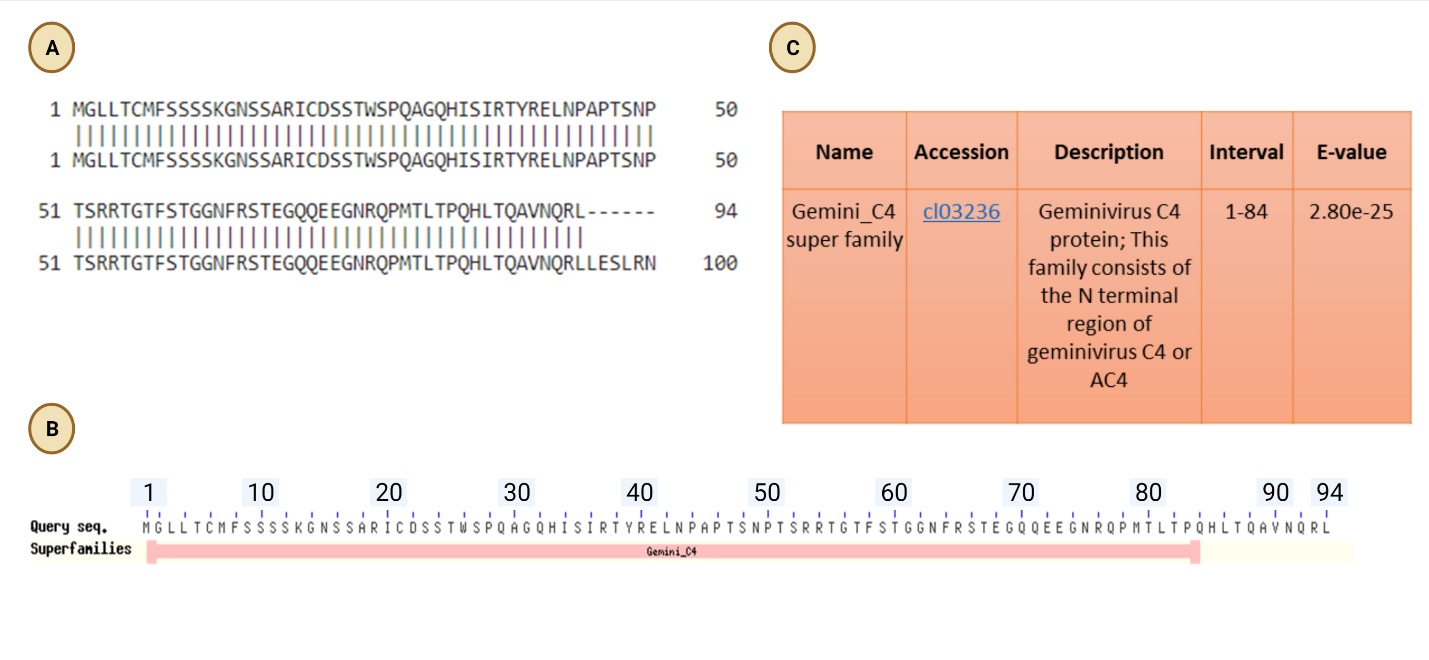


**Fig. S2.** (A) C4 mutated protein (94 aa) was fully aligned with C4 full-length protein (100 aa) leaving back the six amino acids from full-length protein. (B& C) The domains were conserved in both forms of C4 proteins, the domain is Gemini_C4 Superfamily cl03236.

**PSIPRED predicted structures of C1-C1 mutated proteins**


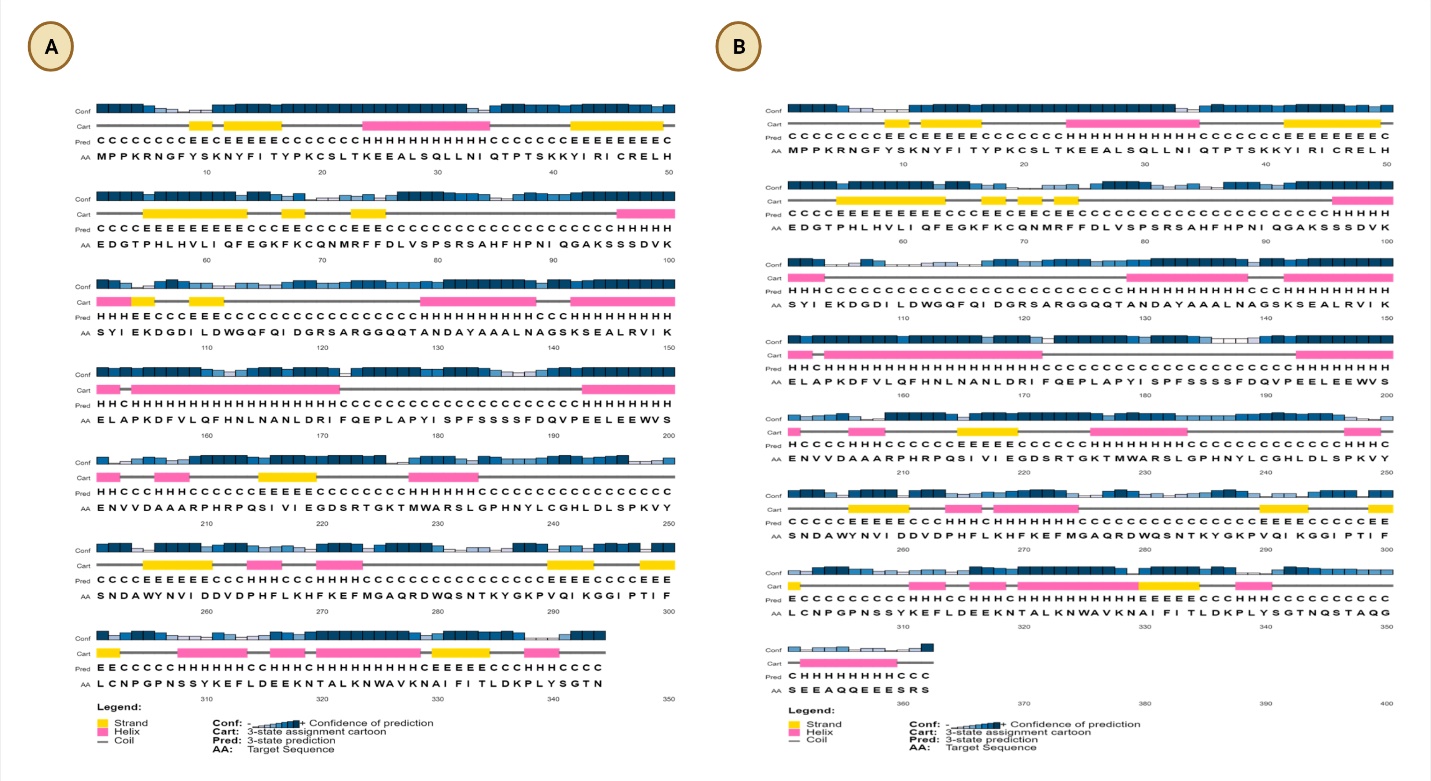


**Fig. S3.** (A) C1 proteins exhibited extra coil and helix region in the full-length protein. (B) but was lacking in mutated C1 protein.

**PSIPRED predicted structures of C4-C4 mutated proteins**


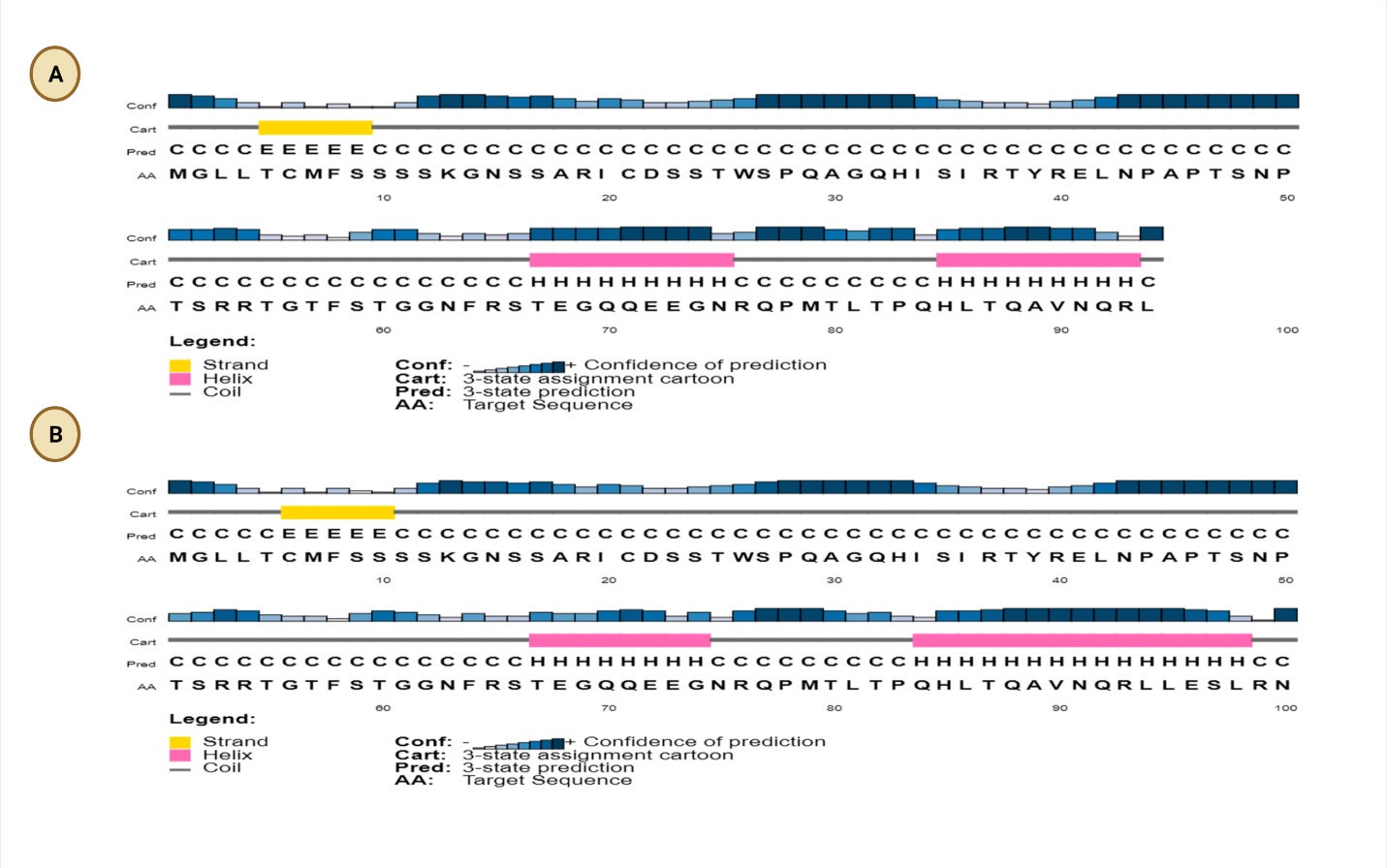


**Fig. S4.** (A) C4 full-length showed one extra coil and six more helix residues, (B) than the mutated C4 partner protein.

**Sequence and motif analysis of C1 proteins**


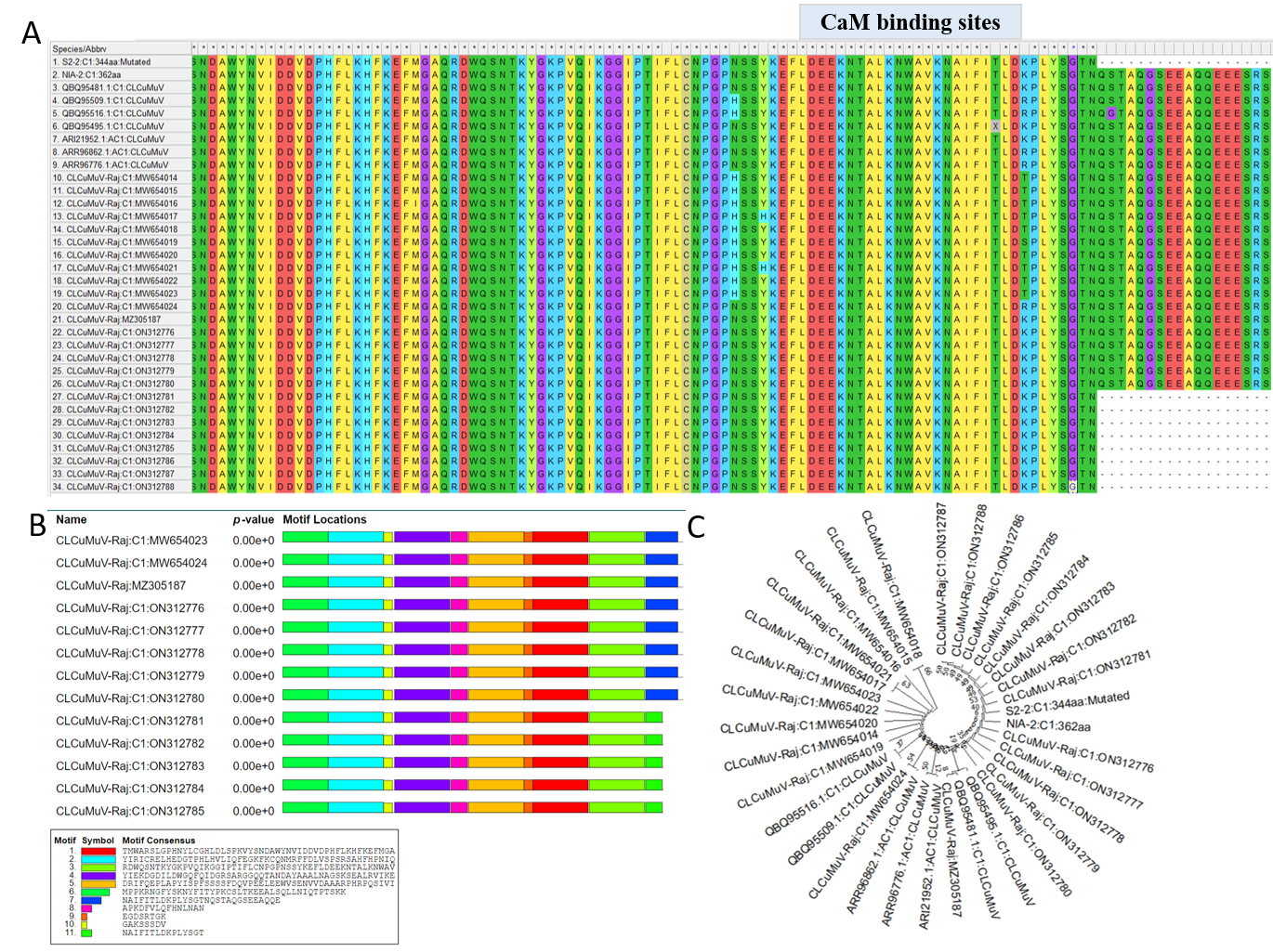


**Fig. S5.** (A) Multiple sequence alignment of C1 protein of isolated strains with reported C1 proteins (B) Conserved de-novo motifs in C1 proteins (C) Evolutionary analysis of C1 mutated proteins with other reported complete proteins.

**Sequence and motif analysis of C4 proteins.**


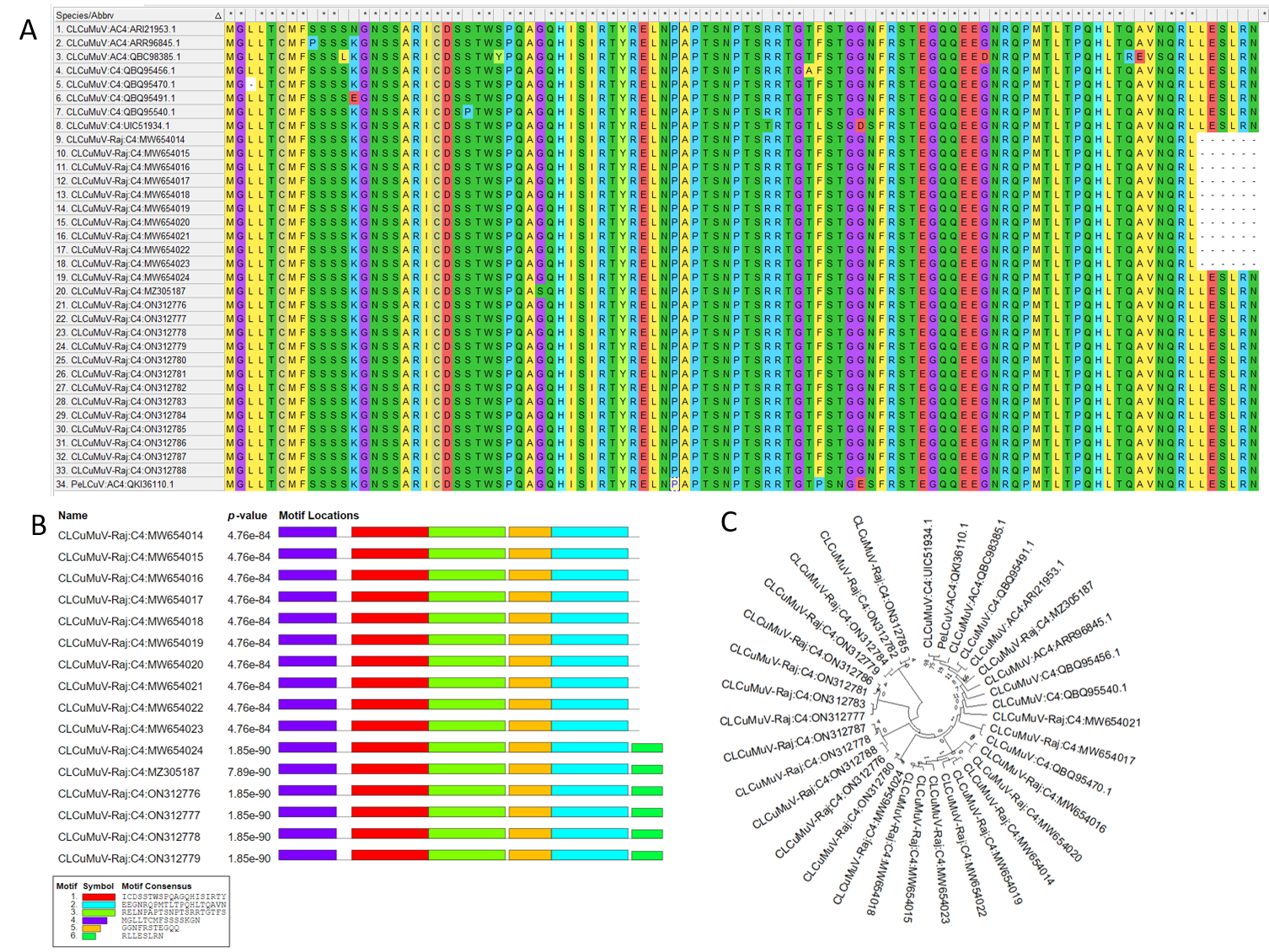


**Fig. S6.** (A) Multiple sequence alignment of C4 protein of isolated strains with reported C4 proteins (B) Conserved de-novo motifs in C4 proteins (C) Evolutionary analysis of C4 mutated proteins with other reported complete proteins.
